# The Decrease of Mineralcorticoid Receptor Drives Angiogenic Pathways in Colorectal Cancer

**DOI:** 10.1371/journal.pone.0059410

**Published:** 2013-03-28

**Authors:** Laura Tiberio, Riccardo Nascimbeni, Vincenzo Villanacci, Claudio Casella, Anna Fra, Valeria Vezzoli, Lucia Furlan, Giuliano Meyer, Giovanni Parrinello, Maurizio D. Baroni, Bruno Salerni, Luisa Schiaffonati

**Affiliations:** 1 Department of Molecular and Translational Medicine, University of Brescia, Brescia, Italy; 2 First Unit of General Surgery, Brescia City Hospital, Brescia, Italy; 3 Department of Pathology, Brescia City Hospital, Brescia, Italy; 4 Department of BioSciences, University of Milano, Milan, Italy; 5 Department of Clinical and Experimental Medicine, University of Padova, Padova, Italy; 6 Department of Biology, University of Padova, Padova, Italy; University of Bari & Consorzio Mario Negri Sud, Italy

## Abstract

Angiogenesis plays a crucial role in tumor growth and progression. Low expression of mineralocorticoid receptor (MR) in several malignant tumors correlates with disease recurrence and overall survival. Previous studies have shown that MR expression is decreased in colorectal cancer (CRC). Here we hypothesize that decreased MR expression can contribute to angiogenesis and poor patient survival in colorectal malignancies. In a cohort of CRC patients**,** we analyzed tumor MR expression, its correlation with tumor microvascular density and its impact on survival. Subsequently, we interrogated the role of MR in angiogenesis in an in vitro model, based on the colon cancer cell line HCT116, ingenierized to re-express a physiologically controlled MR. In CRC, decreased MR expression was associated with increased microvascular density and poor patient survival. In pchMR transfected HCT116, aldosterone or natural serum steroids largely inhibited mRNA expression levels of both VEGFA and its receptor 2/KDR. In CRC, MR activation may significantly decrease angiogenesis by directly inhibiting dysregulated VEGFA and hypoxia-induced VEGFA mRNA expression. In addition, MR activation attenuates the expression of the VEGF receptor 2/KDR, possibly dampening the activation of a VEGFA/KDR dependent signaling pathway important for the survival of tumor cells under hypoxic conditions.

## Introduction

The mineralocorticoid receptor (MR) is a nuclear receptor that has been classically associated to the control of ion transport in epithelial cells, most notably in the kidney and colon. [1], [2] This specific activity plays a crucial role in regulating electrolyte balance and blood pressure. However, it is now known that MR is also expressed in cardiac myocytes, endothelial cells and neurons, suggesting that it plays a physiological role in a large variety of non-epithelial cells. [3], [4] In classical mineralocorticoid target tissues, MR resides mostly in the cytoplasm in an inactive state; upon binding of physiological ligands such as aldosterone, MR undergoes conformational changes, dissociates from molecular chaperones and translocates into the nucleus where it regulates the expression of target genes through specific DNA response elements. [5] There is also evidence of the existence of non-genomic mechanisms, by which activated MR interacts with signaling pathway elements outside the cell nucleus to regulate gene expression. [6] In all cases, the presence of different MR isoforms, the existence of different ligand-induced post-translational modifications of the receptor and the recruitment of different receptor-associated corepressors or coactivators may account for cell-type specific effects of MR within different mineralocorticoid target tissues. [7], [8].

Many literature data indicate that aldosterone, through MR-dependent mechanisms, may also mediate adverse effects on the pathogenesis and progression of ischemic diseases in which angiogenesis plays important role in the rescuing of hypoperfused tissues. [9], [10], [11] Indeed, treatment with MR antagonists promotes a faster and better revascularization reducing the extent of tissue damage in ischemic limb, suggesting that aldosterone, via MR activation, exerts a negative role on angiogenesis. This interpretation is further supported by the finding that in this experimental setting, MR inhibition also correlates with increased expression of pro-angiogenic factors. [12] Moreover, aldosterone impairs vascular regeneration by bone-marrow derived endothelial progenitor cells, a process distinct from angiogenesis, relevant to providing collateral source of blood flow in response to critical narrowing of a major artery [13].

The delineation of molecular mechanisms of adaptive angiogenesis in ischemic tissues has revealed a critical role of the hypoxia-inducible factor-1 (HIF-1) in the transcriptional regulation of genes coding for angiogenic growth factors that mediate the re-growth of the vascular network. [14] In hypoxic cells, the activation of the heterodimeric transcription factor HIF-1 is mainly induced by the lack of the posttranslational modifications of the alpha subunit (HIF-1α) by oxygen-dependent hydroxylase, leading to its rapid degradation under normoxic conditions. As a consequence, under low oxygen concentrations, HIF-1α is stabilized, heterodimerizes with the βsubunit HIF-1β) and binds to hypoxia-response elements (HRE) in target genes [14].

Since a major feature of solid tumors is hypoxia, it is well accepted that tumor elicits an angiogenic response mainly as a result of a HIF-1α-driven increase in angiogenic factor expression, even if dysregulation, due to intrinsic genetic mutations, must also be taken into account. [15] Among different angiogenic growth factors, secreted by both tumor and stromal cells and directly regulated by HIF-1α, VEGFA plays a central role in promoting neovascolarization in cancer. [16] More specifically, VEGFA has been involved in colorectal cancer (CRC) progression, being upregulated in patients with localized as well as metastatic CRC. [17], [18] VEGFA and other members of VEGF family bind to three related membrane receptors (VEGFRs), namely VEGFR1/flt1, VEGFR2/KDR and VEGFR3/flt4, with VEGF receptor 2/KDR playing a pivotal role in mediating cell survival, mitogenesis and differentiation of endothelial cells. VEGF receptor 2/KDR is also expressed on human cancer cells, suggesting it may exert specific roles. [19], [20] Indeed, Calvani and collaborators provided evidence that a VEGF/KDR/HIF-1α autocrine loop mediates survival under hypoxic culture conditions of HCT116, a colon cancer cell line [21].

A previous study reported that a decline of MR expression is an early event in CRC progression and suggested that MR potentially acts as a tumor-suppressor. [22] This notion is consistent with recent reports that showed that, in lung tumors, MR expression levels comparable to those found in normal lung tissue positively correlate with patients overall survival time. [23] In addition, the study in CRC showed that MR underexpression is associated with VEGF receptor 2/KDR overexpression and suggested that underexpression of MR may play a role in the pro-angiogenic switch of the tumor. [22] However to date there has been no mechanistic explanation to this correlation.

In the present study, we first investigated MR expression, angiogenesis and patient survival in a cohort of patients with CRC and demonstrated that decreased MR expression is correlated to increased microvessel density (MVD) and to decreased survival of the patient. We then used an in vitro system based on the colon cancer cell line HCT116, genetically manipulated to express high levels of functionally active MR, to test the hypothesis that MR activation by agonists may negatively regulate tumor angiogenesis. We demonstrated that aldosterone treatment of MR-transfected HCT116 cells decreases the expression of VEGFA mRNA, in both normoxic and hypoxic culture conditions. Moreover, we showed that, in the same cells, aldosterone attenuates the expression of VEGF receptor 2/KDR mRNA.

## Materials and Methods

### Ethics Statement

Thirty consecutive patients undergoing surgery for primary sporadic colorectal cancer were included in the immunohistochemistry and survival analyses. All patients were informed and gave their written consent to the study and the anonymous use of their data before being enrolled. The human study was approved by the Ethics Committee of Spedali Civili of Brescia and the study protocol was in accordance with the Declaration of Helsinki of good clinical practice guidelines.

### MR and CD34 Immunohistochemistry (IHC) and Survival Analysis

Primary endpoints were tumor expression of MR and of CD34, both evaluated by immunohistochemistry. The CD34 endothelial marker was used in order to assess tumor microvessel density (MVD). [24] The following variables were evaluated in order to assess the correlation with the mentioned endpoints: age and gender of patients, colonic site, stage, grade of differentiation, mucinous subtype, and lymphovascular invasion of tumour, intent and setting of primary treatment, overall 5-year survival. Samples of tumour and normal colorectal mucosa were obtained from formaldehyde-fixed surgical specimens. Paraffin sections were stained with hematoxylin-eosin, PAS and PAS Diastase. For the immunohistochemical evaluation the following antibodies were employed: MR (Ab2774, dilution 1∶250 AbCam, Cambridge, UK), after pH6 citrate buffer treatment for 40 minutes and CD34 (CD34Ab1, dilution 1∶30 Thermo Scientific, Astmoor Runcon, UK) after 3 citrate buffer pH 6 cycles. The number of positive cells was counted for each patient in 10 high power fields (HPF, x40) by one pathologist (VV), and the results were categorised into three levels of expression according to the proportion of positive cells; MR expression: low or MR 1+: <33%, intermediate or MR 2+: between 33% and 67%, high or MR 3+: >67%, CD 34 expression: low CD34 1+<33%, intermediate or CD34 2+: between 33% and 67%, high or CD34 3+:>67%.

### Cells

The human colon cancer cell line HCT116 was a kind gift of Dr G Melillo (National Cancer Institute, Frederick, MD, USA) [21]. Wild type HCT116 cells were maintained in RPMI with different concentration of FCS, while transfected HCT116 cells were grown in McCoy’s medium with different concentration of charcoal-stripped FCS or FCS as described below.

### Plasmids

PchMR, which expresses the hMR [25] and pFC31-luc which expresses firefly luciferase under the control of tandem glucocorticoid response element-containing mouse mammary tumor virus promoter [26] were a kind gift from Dr ME Rafestin-Oblin (INSERM U, Paris, France); pRL-TK which expresses the *Renilla* luciferase under the control of herpes simplex virus thymidine kinase promoter was purchased from Promega; pcDNA3 was purchased from Invitrogen.

### Transient Transfection

HCT116 were transfected using FuGENE HD Transfection Reagent (Roche) according to the manufacturer’s instruction. Transfection efficiency for pchMR was assessed at 24h after transfection by immunohistochemistry and found to be 53.5±8%. For an extensive description of this analysis, please see text S1.

### Establishment of Hypoxia

Normoxia was maintained by growing cells at 37°C in a humidified incubator containing 5% CO_2_ in air. Hypoxia was established by maintaining culture flasks at 37°C under a constant flow of an hypoxic gas mixture composed of 95% N_2_ and 5% CO_2_ for 90 min as described. [27] Then flasks were sealed and incubated at 37°C for the desired time of hypoxia.

### Culture and Treatment Conditions of pchMR-MR Transfected HCT116 Cells Grown under Normoxia and Hypoxia

Five hours after transfection with pchMR, HCT116 cells were incubated in Mc Coy’s medium containing 10% hormone-free Charcoal-Stripped Fetal Bovine Serum (Invitrogen) in air for 12 h (normoxia experiments) or 24 h (hypoxia experiments or CoCl_2_ treatment). Cells were then treated with 3 nM aldosterone (Sigma-Aldrich) and/or spironolactone (1 µM, given 1 h prior to aldosterone) (Sigma-Aldrich) and further incubated for 36 h in air (normoxia), or exposed for 20 hours either to lower oxygen (hypoxia) or to 100 µM CoCl_2_ treatment (CoCl_2_). Alternatively, five hours after transfection with pchMR or pcDNA3, cells were incubated in 10% FCS-supplemented medium for 48 hours in air (normoxia) or incubated for 24 hours in air followed by exposure to low oxygen for 20 h (hypoxia).

### Quantitative RT-PCR

RNA was extracted and retrotranscribed as described. [28] Transcript levels were analysed by real-time PCR in an iCycler apparatus (Bio-Rad, Milan, I) with iQ SYBR Green Supermix (Bio-Rad) under conditions recommended by the supplier. PCR Primers, see Table S1, were obtained from Eurofins MWG Operon. Messenger RNA expression levels were normalized to β-actin by the Q-gene software application. [29] Each sample was analyzed in triplicate and PCR products were also separated on 2.5% agarose gel for control.

### Western Blot Analysis

For an extensive description, please see text S1. The following antibodies were used for detection: anti-human MR (dilution 1∶300, kindly donated by Dr Gomez-Sanchez, GV Sonny Montgomery VA Medical Center, Jackson, MS, USA) [30], anti-human HIF-1α (cod. 610658, dilution 1∶800, BD Transduction Laboratories), anti-human GAPDH (sc-32233, dilution 1∶5.000, Santa Cruz).

### Gene Reporter Assay

For an extensive description, please see text S1 in online supplement. PchMR- or pcDNA3- transfected cells were co-transfected with plasmid containing reporter genes. For the detection of MR-driven luciferase expression, pFC31-luc and pRL-TK served as reporter or coreporter gene vector, respectively. Results are given as normalized relative luciferase activity.

### Immunofluorescence

For an extensive description, please see text S1 in online supplement. Fixed cells were incubated with anti-MR antibody (dilution 1∶100) and with Alexa Fluor 448 goat anti-mouse IgG, (Invitrogen). Cell nuclei were counterstained with DAPI. Cells were imaged by the LSM confocal microscope (Zeiss).

### Statistical Analysis

Summary statistics for continuous variables with non-normal distribution (as evaluated by Kolmogorov-Smirnov tests) are presented as medians, while normally distributed variables are summarized as means ± SEM. Group differences were analyzed with Wilcoxon–Mann–Whitney, Student’s t-test or ANOVA followed by Bonferroni t-test as appropriate. The correlation between the expression of MR and CD34 was evaluated by applying Cohen's Kappa and Kramer’s Phi tests. The Kaplan-Meier method and LogRank test were used to examine the effects of different variables on overall survival. No multivariate analysis was performed given the limited sample size and the low ratio of events per variable. The statistical analysis has been performed using the open source statistical package R. All statistical tests were two-tailed and performed at p of 0.05.

## Results

### In Colorectal Carcinoma Patients the Expression of Mineralocorticoid Receptor is Inversely Correlated to Microvessel Density and Poor Prognosis

The baseline clinical characteristics and information on the 5-years follow-up of the patient cohort included in the study are summarised in Table S2.

**Figure 1:**
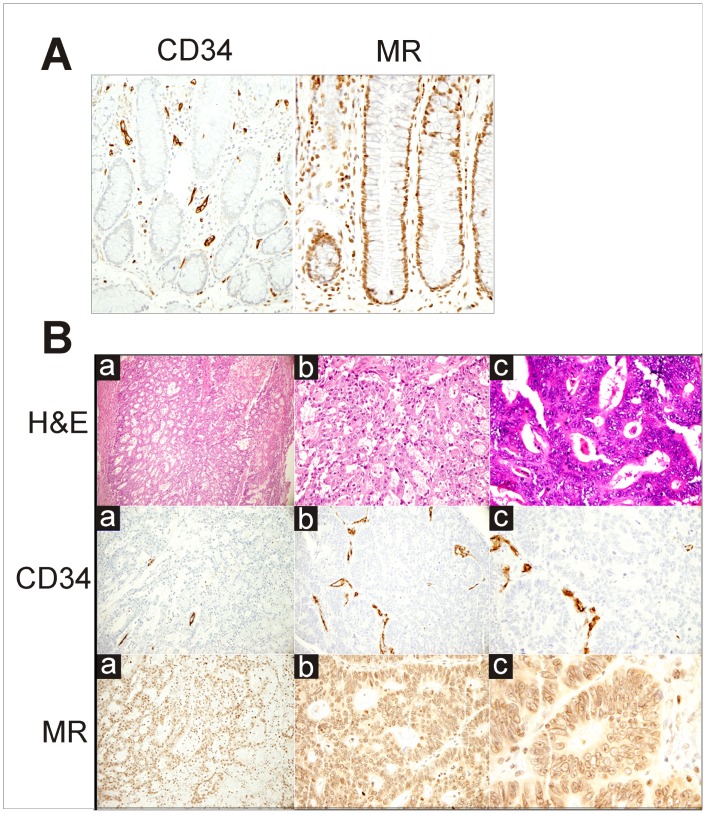
IHC pattern of CD34 and Mineralorticoid Receptor expression in normal colonic mucosa (A) and colonic adenocarcinoma (B) (A, left panel). Scattered vessels positive for CD34 in lamina propria. (x20) and **(A right panel)** Diffuse nuclear positivity for MR in crypts of colonic mucosa. (x40). (**B**) H&E: Adenocarcinoma of the colon, moderately differentiated as shown by Haematoxylin-Eosin staining. CD34: Focal positivity for CD34 in vessels present in the tumor. MR: Diffuse nuclear and cytoplasmatic positivity for MR in tumor cells. Magnification: (a), x10; (b), x20; (c), x40.

Microvessel density, as evaluated by the expression of the endothelial marker CD34, and MR expression were assessed by IHC. A representative pattern of CD34 and MR expression in a CRC sample compared to that of a normal colonic mucosa is shown in Fig. 1B and 1A, respectively. Results from similar analyses in tumor specimens from our patient cohort, based on the determination of the proportion of both CD34 and MR positive cells in the different members of this group, show that CD34 expression was low in 12 subjects, intermediate in 2 subjects, and high in 16 subjects while MR expression was low in 17 subjects, and high in 13 subjects (Fig. 1). There was a significant inverse correlation between tumor expression of CD34 and tumor expression of MR (Kramer Phi coefficient: 0.95, Cohen’s kappa: −0.844, p<0.001). The expressions of both CD34 and MR were associated (directly with p<0.001, and inversely with p<0.001, respectively) with tumor stage categorised into stage I and II as opposed to stage III and IV. Among the other clinico-pathological variables, none was statistically associated to CD34 or MR expression.

**Figure 2:**
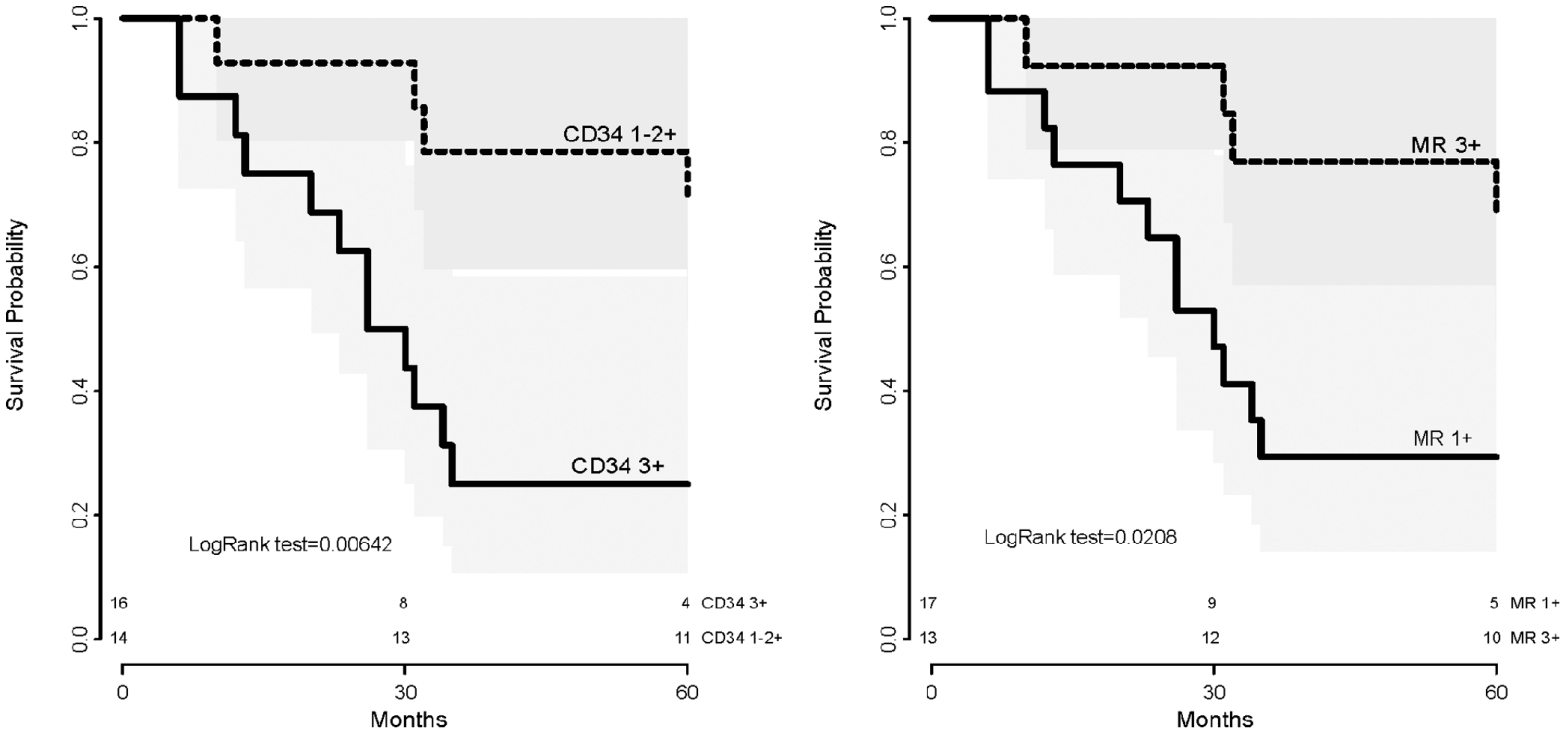
Overall survival stratified by CRC expression of CD34 (left), and CRC expression of MR (right)(Kaplan-Meyer method).

By log rank test, the overall survival was found to be related to the expression of CD34 (p = 0.006), to the expression of MR (p = 0.021) (Fig. 2), and to the intent of treatment (p = 0.002). None of the other clinico-pathologic variables was found to be associated to overall survival, however the impact of tumor stage on survival was at the limit of the range of significance (p = 0.054).

### VEGFA and VEGFR2/KDR Expression is Inhibited by Mineralocorticoid Receptor Activation in a Colon Cancer Derived Cell Line

**Figure 3:**
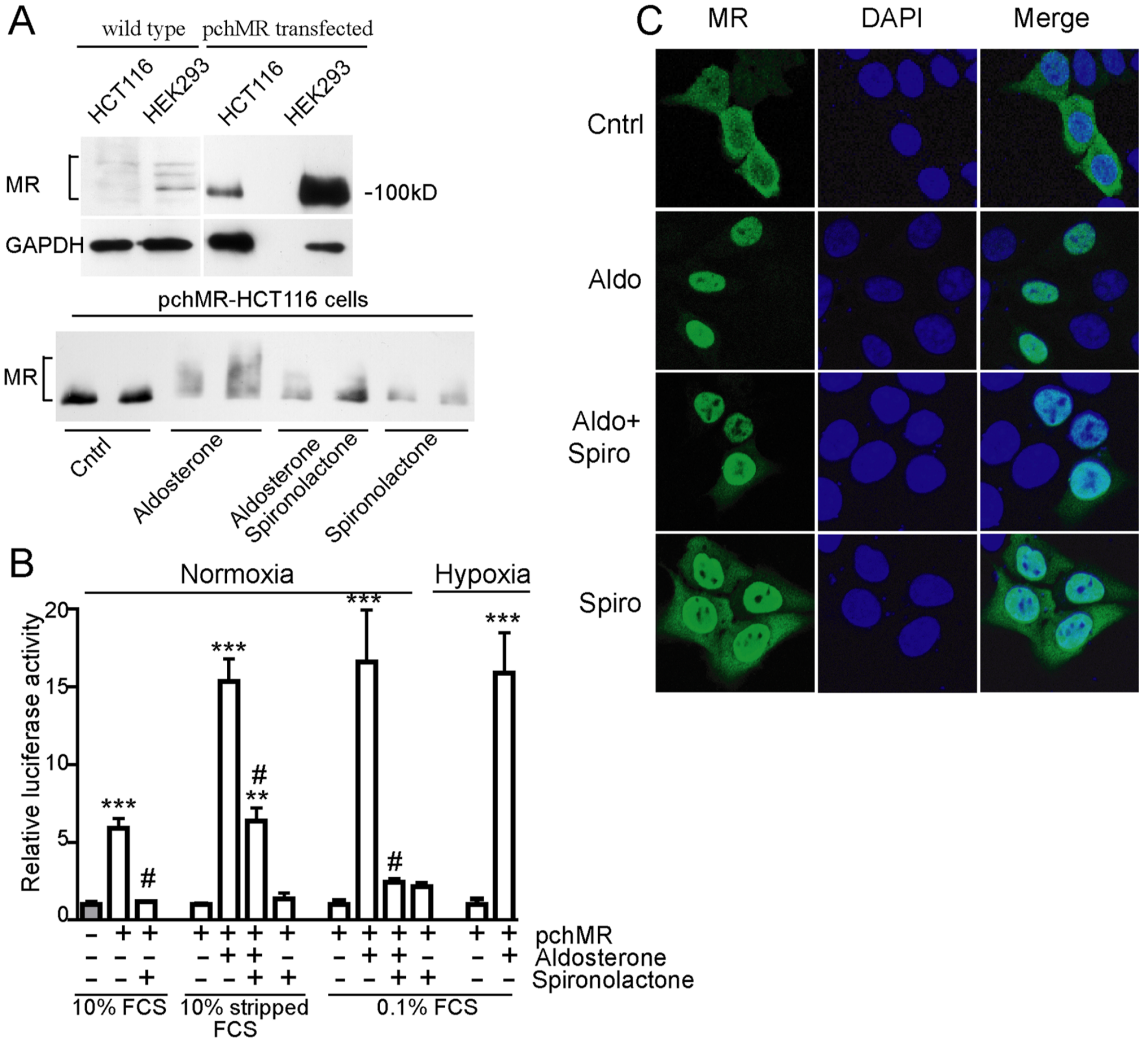
Human mineralocorticoid receptor can be functionally activated in HCT116 cell line. **(A, upper panel)** *MR expression.* Whole cell lysates from wild type and pchMR-transfected HCT116 cells were analysed by western blot using anti-MR antibodies. Human kidney cells (HEK293) served as positive control. Human GAPDH was used as protein loading control. Representative fluorograms from two independent experiments giving similar results are shown **(A, bottom panel)** *MR post-translational modifications.* PchMR-transfected HCT116 cells were treated for 24 h with 3 nM aldosterone and/or 1 µM spironolactone in Mc Coy’s medium with 10% charcoal-stripped FCS. Whole cell lysates were analysed by Western blot using anti-MR antibodies. MR post-translational modifications induced by aldosterone treatment are indicated by the upward shift in the mobility of MR. A representative fluorogram from three independent experiments with superimposable results is shown (**B**) *MR dependent luciferase activity.* PcDNA3-transfected (gray bars) or pchMR-transfected (white bars) HCT116 cells were transfected with pMMTV-Luc to express firefly luciferase from an MR dependent promoter. Cell culture, aldosterone or spironolactone treatment and normoxia or hypoxia conditions are detailed in Materials and Methods section. Values of firefly luciferase activity of aldosterone-stimulated pchMR-transfected cells in 10% stripped FCS or 0.1% FCS, both in normoxic or hypoxic conditions, were compared to those of unstimulated pchMR-transfected control cells, set as 1. Values of firefly luciferase activity of pchMR-transfected cells in 10% FCS were compared to that of pcDNA3-transfected control cells, set as 1. Results were expressed as Mean± SEM (n = 4–6). **p<0.005 and ***p<0.001, vs control cells, ^#^p<0.001 vs FCS- or aldosterone-treated cells, ANOVA followed by Bonferroni t-test or Student t-test when appropriate. (**C**) *MR subcellular localization*. PchMR-transfected HCT116 cells treated with aldosterone (3 nM) and/or spironolactone (1 µM) for 30 minutes and stained with an anti-MR antibody (green) and DAPI (blue). Images were taken with a confocal laser scanning microscope.

To analyse the role played by MR on angiogenesis in CRC and in the light of the important role played by angiogenic factors directly produced by tumor cells in tumorigenesis, we set up an in vitro model by transfecting HCT116, a colon cancer cell line in which endogenous MR protein level was barely detectable (Fig. 3A, upper part), with an efficient MR gene expression system (pchMR plasmid) [25].

We showed that at least 50% of HCT116 cells in culture could be routinely transfected (see *Material and Methods*) and express MR protein at significant levels, as shown by comparing them to HEK293 cells, taken as a positive control (Fig. 3A, upper part). [31] In addition, using different approaches, we showed that in pchMR-transfected HCT116 cells MR is functionally activated by agonists. Indeed, after the supply of aldosterone, MR underwent specific post-translational modifications (Fig. 3A, lower panel), the transcription of a MR reporter plasmid containing luciferase gene was strongly enhanced (Fig. 3B), and MR was translocated into the cell nucleus with the expected kinetics (Fig. 3C). In particular, aldosterone given to the cells grown in medium supplied with either 0.1% or 10% charcoal stripped fetal calf serum significantly enhanced the levels of the luciferase activity (16 folds in aldosterone-treated pchMR-transfected cells vs -untreated controls). It should be noted that luciferase activity was also activated by growing cells in 10% fetal calf serum which naturally contains MR agonists, thus representing a more physiological condition (5 folds in pchMR-transfected versus pcDNA3-transfected cells) (Fig. 3B). [32] Induction of luciferase activity by aldosterone treatment, similar to what found in transfected cells under normoxia, was also found in cells exposed to lower oxygen concentration (Fig. 3B), allowing us to test the effect of MR activation also in cells grown under hypoxic conditions. We also showed that the competitive antagonist spironolactone caused the disappearance of most of the aldosterone induced post-translational modifications while inducing by itself some other specific ones (Fig. 3A lower panel) and largely, though incompletely, abolished the increase of luciferase activity induced by aldosterone (Fig. 3B). Strikingly, the nuclear translocation of MR induced by aldosterone could not be blocked but rather was induced by spironolactone alone (Fig. 3C).

**Figure 4:**
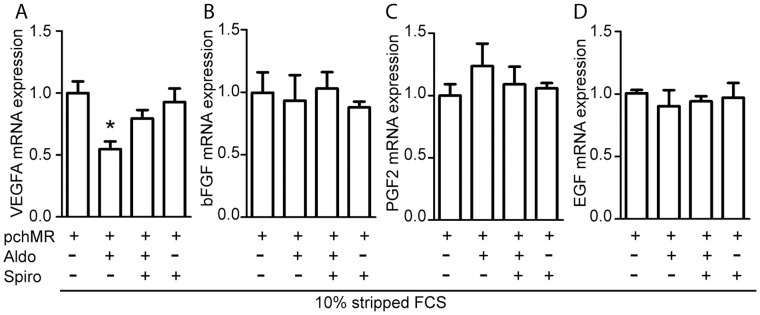
MR activation specifically decreases VEGFA mRNA expression levels in HCT116 cells. Effects of aldosterone on VEGFA (A), bFGF (B), PGF2 (C) and EGF (D) mRNA levels in pchMR-transfected HCT116 cells under normoxic culture conditions. Cells were treated with 3 nM aldosterone in 10% stripped FCS in the absence or in the presence of 1 µM spironolactone and the analysis of mRNA levels were performed by Real-time PCR. For each panel, mRNA expression values of treated pchMR-transfected cells were compared to those of unstimulated pchMR-transfected control cells, set as 1. Results are expressed as Mean±SEM (n = 3). *p<0.05 vs pchMR-transfected control cells, ANOVA followed by Bonferroni t-test.

Functional validation of our cell model allowed us to investigate the possible causal relationship between MR activity and expression changes of mRNA coding for different angiogenic factors both in normoxic and hypoxic environment. We demonstrated that, in pchMR-transfected HTC116 cells grown under normoxic conditions, MR activation by aldosterone induces a significant decrease in VEGF mRNA expression, while it does not affect the mRNA expression levels of other angiogenic factors, namely bFGF, PGF2 and EGF. The aldosterone-induced decrease of VEGFA expression was specifically, albeit partially, inhibited by the competitive MR antagonist spironolactone (Fig. 4). On the whole, these data point out VEGFA as a pro-angiogenic gene potentially regulated by MR activity.

**Figure 5:**
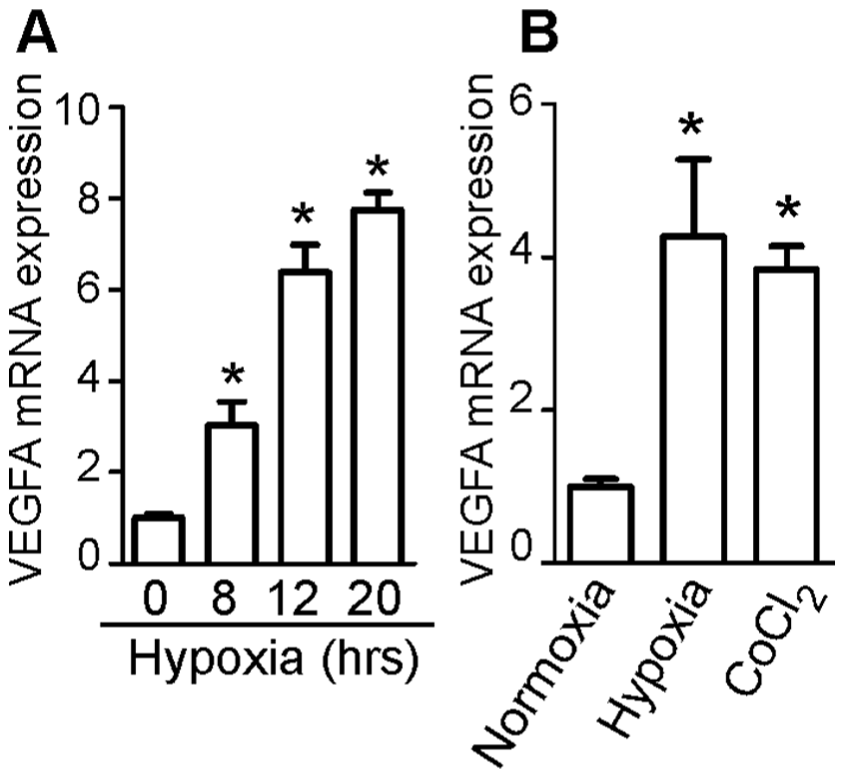
VEGFA mRNA induction during hypoxic response of HCT116 cells. Real-time PCR analysis of VEGFA mRNA induction in wild type HCT116 cells exposed to the indicated times of hypoxia (**A**) and in pchMR-transfected HCT116 cells exposed to normoxia, hypoxia or CoCl_2_ treatment (**B**). In panel A, values of VEGFA mRNA expression for each sample were compared to that of time 0, set as 1. In panel B, values of VEGF mRNA expression of pchMR-transfected cells exposed to hypoxia or CoCl_2_ treatment were compared to that of pchMR-transfected control cells exposed to normoxia, set as 1. Results are expressed as Mean±SEM (n = 3–4). * p<0.05 ANOVA followed by Bonferroni t-test.

**Figure 6:**
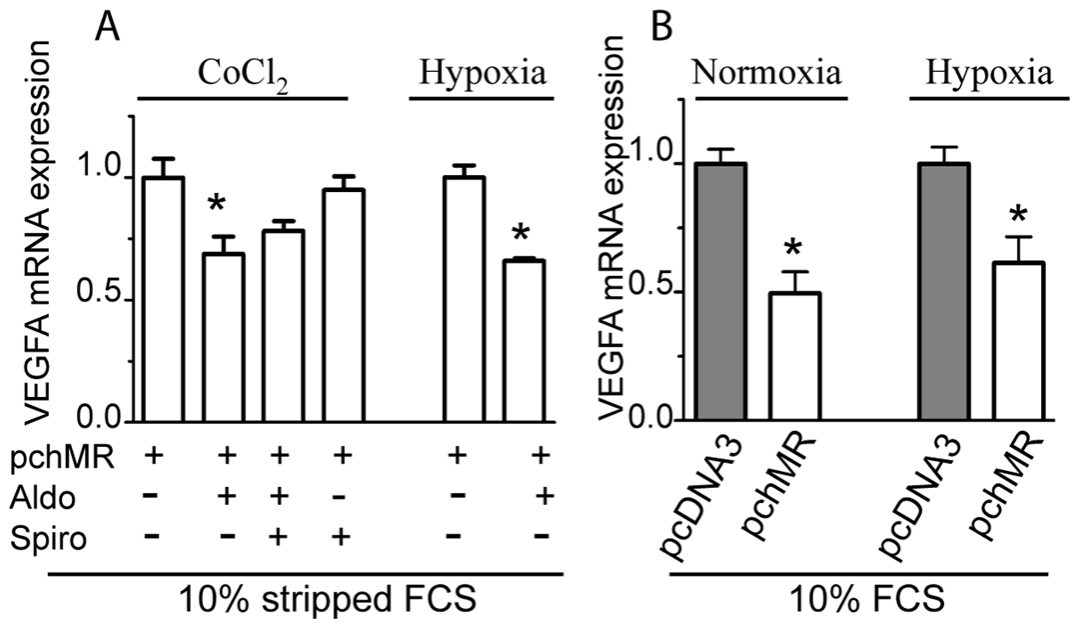
Hypoxia-induced VEGFA mRNA expression is decreased by MR activation in HCT116 cells. Real-time PCR analysis of VEGFA mRNA induction in (A) pchMR-transfected HCT116 cells treated with 3 nM aldosterone in the presence or in the absence of 1 µM spironolactone and exposed to CoCl_2_ treatment or hypoxia and (B) pchMR- or pcDNA3-transfected HCT116 cells cultured with 10% FCS alone under normoxic and hypoxic culture conditions. In panel A, values of VEGFA mRNA expression for each sample were compared to those of unstimulated pchMR-transfected control cells, set as 1. In panel B, values of VEGF mRNA expression of serum-activated pchMR-transfected cells were compared to those of pcDNA3-transfected control cells, set as 1. Results are expressed as Mean±SEM (n = 3–4). *p<0.05 vs pchMR-transfected control cells or pcDNA3-transfected cells, ANOVA followed by Bonferroni t-test or Student t-test when appropriate.

It is well known that hypoxia, a constant characteristic of solid tumor microenvironment, strongly induces VEGFA expression. On these bases we analysed the effect of MR activation on VEGFA expression levels under low oxygen concentration. At this purpose, we first demonstrated that, in pchMR-transfected HCT116 cells, the levels of VEGFA mRNA increase after their exposure to hypoxia similarly to wild type HCT116 cells and that the CoCl_2_ treatment, which mimics hypoxia, strongly increases VEGFA mRNA expression in transfected cells (Fig. 5B and 5A, respectively). We then demonstrated that the decrease of VEGFA expression induced by aldosterone in pchMR-transfected HCT116 cells during normoxia (Fig. 4A) was also found under CoCl_2_ treatment or hypoxic culture conditions (Fig. 6A). These results are particularly significant since we showed that both hypoxia and CoCl_2_ treatment strongly increases VEGFA mRNA expression (Fig. 4B). Moreover, we also show that MR activation by the agonists naturally present in whole FCS is able to decrease VEGFA mRNA expression both under normoxic and hypoxic conditions (Fig. 6B ).

Finally, on the bases of recent reports by Calvani and collaborators on the role of KDR expressed in HCT116 cells in sustaining cell survival after exposure to prolonged hypoxia, we analyzed, in pchMR transfected HCT116 cells, a possible causal relationship between MR activity and KDR expression changes. Indeed, these authors demonstrated that KDR mediates a late VEGF-dependent induction of HIF-1α, leading to the autocrine production of VEGFA, since they could inhibit both HIF-1α-induction and survival of hypoxic HCT116 cells, with either anti-VEGFA or anti-KDR antibodies. [21] Thus, as a preliminary experiment, we demonstrated the presence of a functional VEGF/KDR/HIF1α autocrine loop in our HCT116 cell line, by reproducing the lack of the late induction of HIF-1α by VEGFA antibodies in cells grown under hypoxic conditions (Fig. S1).

**Figure 7:**
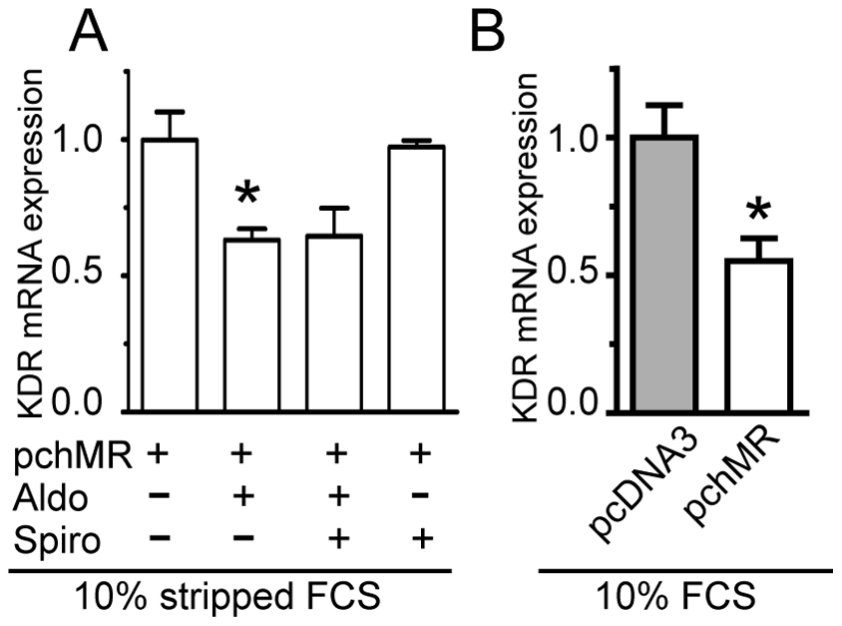
MR activation decreases KDR expression levels in HCT116 cells. *Effects of aldosterone* (**A**) *or serum* (**B**) *on KDR mRNA levels.* Cell culture and treatments conditions were as in figure 4, or figure 6 panel B-Normoxia, respectively. Analyses of KDR mRNA levels were performed by Real-time PCR and results (n = 5) are given as in Fig 5. *p<0.05 vs pchMR-transfected control cells or pcDNA3-transfected cells, ANOVA followed by Bonferroni t-test or Student t-test when appropriate.

We then demonstrated that, in pchMR-transfected HCT116 cells, MR activation induced a significant decrease in the levels of KDR mRNA. KDR mRNA expression was decreased in aldosterone stimulated pchMR-transfected HCT116 cells to about 65% respect to their unstimulated controls (Fig. 7A) and even to a greater extent in serum stimulated pchMR- transfected HTC116 compared to pcDNA3–transfected controls (Fig. 7B). Strikingly, although spironolactone did not significantly modify KDR expression levels, it appeared to reverse only in part the effects of aldosterone treatment in pchMR-transfected HCT116 cells. Indeed, even if a similar decrease in KDR expression was observed in aldosterone- and spironolactone-aldosterone-treated cells as compared to controls, in the latter case the decrease was not statistically significant (Fig. 7A). Reasons that may account for different spironolactone potency in reversing the effects elicited by active MR on different targets or in different contexts will be discussed below.

## Discussion

Because previous studies have shown that MR expression is down regulated in both colorectal and lung cancers, it has been suggested that MR may act as a tumor-suppressor gene [23].

Here we establish a link between underexpression of MR, decreased patient’s survival and upregulation of tumor angiogenesis in advanced cancer stage. Using an in vitro model based on a colon carcinoma cell line, in which we forced MR expression, we also provide the evidence that activated MR can attenuate the expression of VEGFA and its receptor 2/KDR.

A link between MR expression and angiogenesis in CRC has been previously suggested. [22] Here we demonstrate that the extent of MR positive cells is inversely correlated to MVD in tumor specimens, supporting the hypothesis that decreased MR expression releases a repressing role exerted by MR on tumor angiogenesis. To give insights on the role played by MR in CRC angiogenesis, we showed that the re-expression of activated MR in a colon cancer cell line, characterized by a quite low MR protein level, thus mimicking a key feature present in CRC in vivo, leads to a specific decrease in mRNA expression of VEGFA among other angiogenic factor analyzed, in cells under normoxic culture conditions. These data provide a direct demonstration of a suppressive role of MR in tumor angiogenesis driven by the malignant epithelium. It is noteworthy that our findings in colon cells are consistent with the results of a recent study in a transgenic mouse model showing that long-term *in vivo* MR overexpression, in the presence of physiological amount of aldosterone, specifically downregulated VEGFA gene expression in the heart [33].

Little is known about the regulation of angiogenic growth factors in tissue under normoxic conditions. However it is well accepted that physiological stimuli, other than hypoxia, including growth factor activated signaling pathways, can also induce HIF-1α activation and the consequent transcription of hypoxia-inducible genes under non hypoxic conditions. [34] In addition many genetic alterations present in cancer cells can directly increase HIF-1α expression, leading to the activation of VEGFA gene expression, independently from intratumoral hypoxia. [14], [35] These data provide the molecular mechanisms linking specific genetic alterations present in cancer cells with increased tumor vascularization. Based on these literature data and on our results from the analysis of VEGFA mRNA expression in MR-transfected colon cancer cells grown under normoxic conditions upon activation by the relative agonists, we suggest that MR may inhibit deregulated angiogenesis in CRC.

However, here we suggest that activated MR also dampens hypoxia-regulated angiogenesis, which is crucial for tumor cells to survive and proliferate in a hostile microenvironment and is a critical determinant of the tumor progression. [36] Indeed we demonstrated that re-expressed and agonist-activated MR reduced VEGFA mRNA expression in colon cancer cells even when maximally activated by their exposure to lower oxygen concentration or CoCl_2_ treatment.

In addition, our findings that, in MR-transfected colon cancer cells, KDR expression was significantly decreased by MR activation indicated that activated MR can inhibit also the expression of the receptor 2 of VEGFA, thus strengthening the hypothesis of a causal relationship between MR underexpression and KDR overexpression found in CRC by Di Fabio and collaborators. [22] On the basis of their results, these authors hypothesized that MR underexpression may play a role in the proangiogenic switch of the tumor through the enhancement of KDR expression. Here we suggest mechanistic insights on the role of increased KDR in promoting colorectal tumorigenesis, other than supporting angiogenesis. Indeed, it has been shown that VEGFA binds to KDR expressed on colon cancer cells and, through activation of specific KDR downstream signaling pathways, leads to a late induction of HIF-1α, which in turn mediates an autocrine production of VEGFA. This latter then acts as an important survival factor in colon cancer cells when cultured under conditions which mimics oxygen deprivation found in solid tumors. [21] The involvement of VEGFA in mediating survival of hypoxic cancer cells was surprising because VEGFA, mainly produced by stromal infiltrating cells or by tumor cells and acting in a paracrine way, was thought to be primarily a survival factor for endothelial cells. [16] It is noteworthy, that a similar feed-back mechanism of hypoxic response, based on an HIF-1α-driven VEGFA-mediated autocrine loop, has been reported also in endothelial cells and shown to exert an autonomous control on chemotaxis, mitogenesis and survival of endothelial cells, thus directly contributing to neo-vascularization in hypoxic tissues [37].

Strikingly our results on the role of activated MR in the attenuation of the expression of KDR in MR-transfected colon cancer cells, agree with similar data obtained in endothelial progenitor cells and HUVEC. [13], [38] When compared with our results, the data obtained in HUVEC showed that KDR mRNA was similarly down-regulated by aldosterone, although the reduction was less pronounced (30% vs 40%) even if they used a higher concentration of aldosterone (10 nM vs 3 nM).

An unexpected result in our study is the only very partial efficacy of the competitive MR antagonist spironolactone in reversing the repressive effect of aldosterone on the expression of both VEGFA and its receptor KDR. Indeed, the quite similar inhibitory effects of aldosterone seen in HUVEC were reversed to the basal level with 10 µM eplerenone. [38] Beyond the obvious differences related to the cellular systems and MR antagonists (and their concentration), there are several possible explanations of this discrepancy. First, in all tested *in vitro* systems spironolactone effects counteracting MR activation appear to be virtually partial, varying as a function of cells, protocols and process under investigation. Second, the unique western blot signal pattern of MR seen when spironolactone is given together with aldosterone prompted us to speculate that the receptor functional activity cannot be fully comparable to a negative control. The result of one set-up experiment of this study is consistent with this view, since spironolactone could not completely abrogate the aldosterone induced luciferase increase. Since we kept fixed any parameter in the other set-up tests but the culture conditions, these latter ones also appear to influence the degree of spironolactone reversion. Finally, aldosterone can produce rapid non genomic effects that are basically insensitive to spironolactone. These are mediated by classical MR associated to a membrane complex and, likely, a G-protein coupled membrane receptor. [39] We do not know if the fraction of MR kept in the HCT116 cytoplasm upon aldosterone addition is simply a side effect of receptor overexpression or it does have a functional meaning out of the nucleus. Other inhibitors, such as RU28318, are needed to inhibit these membrane associated complexes and could be tested to address this particular item [40].

In conclusion, our in vivo and in vitro studies allowed us to demonstrate that MR can negatively regulate colorectal tumorigenesis. Using an original in vitro model based on a colon cancer cell line *ad hoc* ingenierized to express high levels of agonist-regulated MR, we showed that the expression of an active MR is causally linked to a decrease in the expression of VEGFA, which sustains angiogenesis, critical for growth and progression of solid tumors in a hypoxic environment. In addition, we showed that the expression of an active MR is causally related to the inhibition of KDR expression, possibly leading to the dampening of a specific VEGFA/KDR signal transduction pathway, driving angiogenesis in endothelial cells under hypoxic condition, but exploited by colon cancer cells to survive in a hypoxic environment.

## Supporting Information

Figure S1 HIF-1α protein increases during hypoxic response of HCT116 cells. Wild type HCT116 cells were cultured in RPMI with 0.1% FCS and exposed to a hypoxia lasting 20 hours in the presence or absence of 200 ng/ml of anti-VEGFA antibodies. Whole cell lysates were analysed by Western blot using anti-HIF-1α antibodies. GAPDH was used as protein loading control. Representative fluorograms from three independent experiment are shown.
(TIF)

Table S1 PCR primers.
(DOC)

Table S2 Baseline characteristics of patients included in immunohistochemistry study.
(DOC)

Text S1 Materials and Methods.
(DOCX)
