# Supplementary figures and images for: The Decrease of Mineralcorticoid Receptor Drives Angiogenic Pathways in Colorectal Cancer
Source: PLoS One. 2013 Mar 28;8(3):e59410. doi: 10.1371/journal.pone.0059410 (PMC3610652; doi:10.1371/journal.pone.0059410)

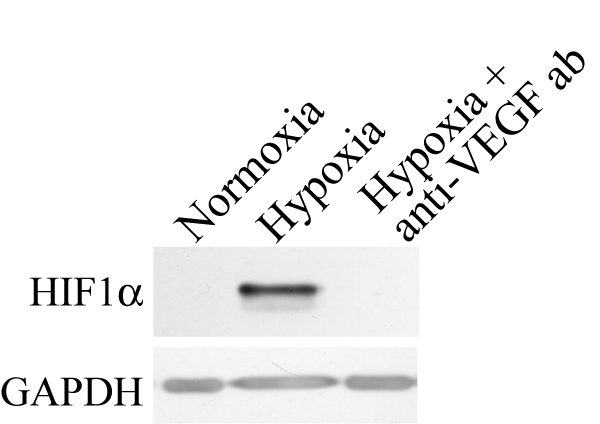

Supplement: Figure S1 — HIF-1α protein increases during hypoxic response of HCT116 cells. Wild type HCT116 cells were cultured in RPMI with 0.1% FCS and exposed to a hypoxia lasting 20 hours in the presence or absence of 200 ng/ml of anti-VEGFA antibodies. Whole cell lysates were analysed by Western blot using anti-HIF-1α antibodies. GAPDH was used as protein loading control. Representative fluorograms from three independent experiment are shown. (TIF) [file pone.0059410.s001.tif]
