# Supplementary material for: The Decrease of Mineralcorticoid Receptor Drives Angiogenic Pathways in Colorectal Cancer
Source: PLoS One. 2013 Mar 28;8(3):e59410. doi: 10.1371/journal.pone.0059410 (PMC3610652; doi:10.1371/journal.pone.0059410)
Supplement: Table S1 — PCR primers. (DOC) [file pone.0059410.s002.doc]

**Supplementary table S1:**  PCR primers

| MR, forward | 5’-GAGGCTTCAGGATGCCATTA-3’ |
| --- | --- |
| MR reverse | 5’-GCTCCTCGTGAATCCCTTTT-3’ |
| VEGFR-2, forward | 5’-GGTGTTTTGCTGTGGGAAAT-3’ |
| VEGFR-2 reverse | 5’-AAACGTGGGTCTCTGACTGG-3’ |
| bFGF, forward | 5’-CTGGCTATGAAGGAAGATGGA-3’ |
| bFGF reverse | 5’-TGCCCAGTTCGTTTCAGTG-3’ |
| VEGF-A, forward | 5’-AGTGTGTGCCCACTGAGGA-3’ |
| VEGF-A reverse | 5’-GGTGAGGTTTGATCCGCATA-3’ |
| PGF2, forward | 5’-TGCAGCTCCTAAAGATCCGTTC-3’ |
| PGF2 reverse | 5’-GTGGCAGTCTGTGGGTCTCT-3’ |
| β-Actin, forward | 5’-TCTTCATTGTGCTGGGTGCC-3’ |
| β-Actin, reverse | 5’-TGACGTGGACATCCGCAAAG-3’ |
