# Supplementary material for: The Decrease of Mineralcorticoid Receptor Drives Angiogenic Pathways in Colorectal Cancer
Source: PLoS One. 2013 Mar 28;8(3):e59410. doi: 10.1371/journal.pone.0059410 (PMC3610652; doi:10.1371/journal.pone.0059410)
Supplement: Table S2 — Baseline characteristics of patients included in immunohistochemistry study. (DOC) [file pone.0059410.s003.doc]

**Supplementary Table S2:**

Baseline characteristics of patients included in immunohistochemistry study

| **Clinical /Pathological characteristics** |  |
| --- | --- |
| Age at surgery, median years (range) | 65 years (56-71) |
| Gender, no. males (%) / no. females (%) | 22(73) / 8(27) |
| Tumour site, no. (%)  *Right colon*  *Left colon*  *Rectum* | 8 (27)  19 (63)  3 (10) |
| Tumour UJCC stage, no. (%)  *Stage I*  *Stage II*  *Stage III*  *Stage IV* | 1(3)  11(37)  13 (43)  5 (17) |
| Grade of differentiation, no. (%)  *Grade 2*  *Grade 3* | 24 (80)  6 (20) |
| Mucinous subtype, no. (%) | 2 (7) |
| Lymphovascular invasion, no. (%) | 18 (60) |
| Curative intent of treatment, no. (%) | 22 (73) |
| Emergency surgery, no. (%) | 3 (10) |

No patient was lost from follow-up. Five years after surgery 13 patients (43%) were alive and disease free, 2 (7%) were alive with disease, 2 (7%) were deceased due to unrelated diseases, and 13 (43%) were deceased of metastatic colorectal cancer.
