# Supplementary material for: The Decrease of Mineralcorticoid Receptor Drives Angiogenic Pathways in Colorectal Cancer
Source: PLoS One. 2013 Mar 28;8(3):e59410. doi: 10.1371/journal.pone.0059410 (PMC3610652; doi:10.1371/journal.pone.0059410)
Supplement: Text S1 — Materials and Methods. (DOCX) [file pone.0059410.s004.docx]

**Supplementary text S1: Materials and Methods**

**Transient Transfection**

For this analysis, cells were fixed in paraformaldehyde 4%, permeabilized in PBS/Triton 0.1%, blocked in Horse Serum (Vector Laboratories), incubated for 2h at room temperature with the anti-human MR antibody (mouse monoclonal, 6G1-3E3, used at the dilution of 1:50,(31)) followed by biotin-conjugated anti-mouse IgG, and finally developed using the Vectastain ABC Kit (Vector Laboratories) and 3,3'-Diaminobenzidine staining. MR-expressing cells were scored in 22 randomly selected fields containing about 150 cells per field.

**Western blot analysis**

Cells were scraped from the dishes and lysed in ice-cold Lysis Buffer (10 mM TRIS pH 7.4, 150mM NaCl, 1% NP40). Protein Inhibitor Cocktail (Sigma-Aldrich), and 1 mM NaVO_3_ were always added. Lysates were centrifuged at 14000 rpm for 10 min at 4°C and protein content of supernatants were determined by Bio-Rad Protein Assay (Bio-Rad Laboratories, Milan, Italy). Total cell protein extracts were resolved by SDS-PAGE (using different acrylamide-bis-acrylamide concentration i.e. 7% for MR, 8% for HIF1α and 12% for GAPDH), transferred to PVDF Membrane (Millipore), and incubated overnight at 4°C with the appropriate primary antibodies. The membranes were then incubated with peroxydase-conjugated anti-mouse antibody (GE Healthcare) and immunoreactive proteins were visualized by ECL Plus (GE Healthcare). Western blot analysis using anti-GAPDH (Glyceraldehyde-3-phosphate dehydrogenase) was performed as a control for protein loading.

**Gene reporter assay**

For experiments performed under normoxia, transfected cells were incubated for 24h in a medium containing 0.1% FCS or 10% charcoal-stripped FCS in the presence of 3 nM aldosterone and /or of 1 µM spironolactone or in a medium containing 10% FCS in the presence or in the absence of 1 µM spironolactone. When added, spironolactone was given 1 hour prior to aldosterone. For the experiments performed under hypoxic conditions, transfected cells, maintained in a medium containing 0.1% FCS, were left untreated for 24h and then exposed to the hypoxic gas mixture for 90 min. Subsequently, hypoxic cells were stimulated with 3nM aldosterone and further incubated for 24h. Treated cells were then lysed with passive lysis buffer (Promega) and both firefly and *Renilla* luciferase expression was monitored with the Dual-Luciferase Reporter Assay System (Promega) using the manufacturer's approved reagents and protocols and the GloMax^®^ 20/20 Luminometer.

**Immunofluorescence**

Cells were plated on sterile glass coverslips and transfected with pchMR vector. At five hours after transfection, cells were incubated in McCoy’s medium with 0.1% FCS for 24 hours and then treated with aldosterone (3nM) and/or spironolactone (1µM) for 30 minutes. Cells were fixed with 4% paraformaldheyde, permeabilized with 0.5% Triton X-100 in PBS, blocked with 5% normal goat serum (Dako) in PBS, and incubated with anti-MR primary antibodies for 2 hours at room temperature followed by Alexa Fluor 488 conjugated goat anti-mouse IgG (dilution 1:500). Nuclei were counterstained with DAPI (1:1000 Roche). Coverslips were mounted with glycerol, and imaged by the LSM confocal microscope (Zeiss).
